# Effect of a nurse-led lifestyle choice and coaching intervention on systolic blood pressure among type 2 diabetic patients with a high atherosclerotic cardiovascular risk: study protocol for a cluster-randomized trial

**DOI:** 10.1186/s13063-021-05085-z

**Published:** 2021-02-11

**Authors:** William Lumu, Davis Kibirige, Ronald Wesonga, Silver Bahendeka

**Affiliations:** 1 Department of Internal Medicine, Mengo Hospital, Kampala, Uganda; 2 Uganda Martyrs Hospital Lubaga, Kampala, Uganda; 3 East African Statistics Institute (EASI), Kampala, Uganda; 4 Mother Kevin Post Graduate Medical School, Uganda Martyrs University, Nkozi, Uganda

**Keywords:** Nurse-led lifestyle choice, Coaching intervention, Systolic blood pressure, Atherosclerotic cardiovascular risk, Type 2 diabetes, Randomized controlled trial

## Abstract

**Background:**

More than 50% of patients with type 2 diabetes have hypertension in Uganda. Diabetic patients with elevated systolic blood pressure experience higher all-cause mortality and cardiovascular events compared with normotensive diabetic individuals, hence escalating resource utilization and cost of care. The aim of this study is to determine the effect of a nurse-led lifestyle choice and coaching intervention on systolic blood pressure among type 2 diabetic patients with a high atherosclerotic cardiovascular risk.

**Methods:**

This is a cluster-randomized study comprising two arms (intervention and non-intervention—control arm) with four clusters per arm with 388 diabetic patients with a high predicted 10-year atherosclerotic cardiovascular risk. The study will be implemented in 8 health facilities in Uganda. The intervention arm will employ a nurse-led lifestyle choice and coaching intervention. Within the intervention, nurses will be trained to provide structured health education, protocol-based hypertension management, and general atherosclerotic cardiovascular risk factor management, 24-h phone calls, and 2-monthly text messaging. The control group will be constituted by the usual care. The primary outcome measure is the mean difference in systolic blood pressure between the intervention and usual care groups after 6 months. The study is designed to have an 80% statistical power to detect an 8.5-mmHg mean reduction in systolic blood pressure from baseline to 6 months. The unit of analysis for the primary outcome is the individual participants. To monitor the effect of within-cluster correlation, generalized estimating equations will be used to assess the changes over time in systolic blood pressure as a continuous variable.

**Discussion:**

The data generated from this trial will inform change in the policy of shifting task of screening of hypertension and atherosclerotic cardiovascular disease from doctors to nurses.

**Trial registration:**

Pan African Trials Registry PACTR 202001916873358. Registered on 6 October 2019

## Administrative information

The order of the items has been modified to group similar items (see http://www.equator-network.org/reporting-guidelines/spirit-2013-statement-defining-standard-protocol-items-for-clinical-trials/).

**Table:** 

Title {1}	Effect of a nurse-led lifestyle choice and coaching intervention on systolic blood pressure among Type 2 diabetic patients with a high atherosclerotic cardiovascular risk: study protocol for a cluster randomized trial
Trial registration {2a and 2b}.	Pan African Trials Registry; PACTR 202001916873358. Item 2b is met.
Protocol version {3}	Version 03, 2021 Jan 19
Funding {4}	No source of funding to declare.
Author details {5a}	William Lumu^1^ (corresponding author) Department of Internal Medicine Mengo Hospital Kampala Uganda P.O Box 7161 Kampala Email dhabaguma@gmail.com +256772591911 Davis Kibirige^2^ Uganda Martyrs Hospital Lubaga Kampala Uganda Email kibirigedavis@gmail.com Ronald Wesonga^3^ Makerere University, School of Statistics and Planning Email wesonga@wesonga.com Silver Bahendeka^4^ Mother Kevin Post Graduate Medical School-Uganda Martyrs University, Uganda Email silverbahendeka@gmail.com bahendeka@yahoo.com
Name and contact information for the trial sponsor {5b}	No sponsor for the trial
Role of sponsor {5c}	This is an investigator led trial. William Lumu, the Principal Investigator is involved in study design; collection, management, analysis, and interpretation of data; writing of the report; and the decision to submit the report for publication.

## Introduction

### Background and rationale {6a}

By 2040, it is estimated that 642 million people will have type 2 diabetes worldwide while 1.39 billion people already have hypertension [1].

In Sub-Saharan Africa, hypertension affects younger age groups than in the developed world and is the major risk factor for heart failure, stroke, and kidney failure [2] This risk increases when one has diabetes.

In Uganda, the prevalence of hypertension is high ranging from 11 to 32% [3–7]. In a nationwide STEPS survey covering all major regions of the country with 3906 participants, the prevalence of hypertension was 26.4% [8].

More than 50% of patients with type 2 diabetes have hypertension [9].

Both conditions orchestrate cardiovascular disease [1, 2, 8]. There is a bi-directional relationship between diabetes and hypertension as patients with hypertension have insulin resistance and are at risk of developing diabetes in their lifetime [1].

The two conditions share similar pathophysiological pathways, namely endothelial dysfunction, vascular inflammation, arterial remodeling, atherosclerosis, dyslipidemia, and obesity [1, 9] Further analysis of Framingham data showed higher all-cause mortality, cardiovascular events in hypertensive diabetic patients compared with normotensive diabetics resulting from excessive atherosclerotic cardiovascular disease(ASCVD) risk [10].

A combination of diabetes and hypertension portends higher costs and resource utilization as this patient cohort suffers more myocardial infarctions and acute ischemic events, thus escalating the cost of care [9, 11]. In the USA, in a study by the National Health and Nutritional Survey (NHANES) 2005–2008, only 50% of hypertensive individuals had their blood pressure under control [12].

In Sub-Saharan Africa, the majority of patients have poorly controlled hypertension resulting from poor access to care and shortage of physicians [13].

In all the prevalence studies in Uganda, there are low rates of awareness of the condition among the patients [5, 6, 8, 14], resulting in low rates of treatment [4, 5], and control [2] leading to hypertension-related cardiovascular disease.

Among diabetic individuals, early landmark trials have demonstrated the benefits of strict BP control as it is associated with a reduction in heart failure and myocardial infarction (United Kingdom Prospective Diabetes Study (UKPDS) [15], Hypertension Optimal Treatment (HOT) [16], Systolic Hypertension in the Elderly (SHEP) [17], and Systolic Hypertension in Europe (Syst-Eur) [18]) though there is still controversy on the blood pressure target among type 2 diabetic patients.

Additionally, decreasing systolic blood pressure by 10 mmHg results in a 12% reduction of diabetes-related complications [19, 20].

Despite sufficient evidence for the benefit of hypertension control among type 2 diabetes, there is suboptimal control of blood pressure. In one systemic review of observational studies on treatment and blood pressure control in 47,964 people with diabetes and hypertension, control of blood pressure in patients with diabetes was suboptimal with less than 12% of patients achieving a blood pressure target of 130/80 mmHg [21].

In Sub-Saharan Africa, poor access to health care and physician shortage are the major hindrances to the control of hypertension [13].

In Uganda, control of hypertension among diabetic patients is still suboptimal as only 56% of them have their BP ≤ 140/80 mmHg [22].

A leading cause of suboptimal control of hypertension in Uganda is the lack of screening among patients with diabetes [22].

Other barriers to good blood pressure control are limited resources, ill-equipped health facilities, scarce medications, inadequate training of personnel, insufficient knowledge and experience of clinicians in the management of cardiovascular disease and hypertension, and lack of guidelines [23].

Diabetes and hypertension share similar risk factors such as insulin resistance, obesity, dyslipidemia, and endothelial dysfunction [1]. Effective control of both conditions requires a multifactorial approach. The STENO 2 trial demonstrated the benefit of multiple ASCVD risk factor management [24, 25].

Therefore, an intensive program that involves counseling about medical nutrition therapy, physical activity, smoking cessation, medication adherence, and behavior change with ongoing support and frequent follow-up are key for lifestyle management of weight, dyslipidemia, hypertension, and glycemia in patients with type 2 diabetes [25].

In the primary care setting, management of ASCVD and hypertension is done by doctors, but these are few [13] and spend less time with the patients; hence, there is a need to shift this task to other health care cadres such as nurses. There is evidence that nurses are traditionally well versed in self-care support and play a leading role in the administration of systematic educational interventions [26].

A systematic review of studies of nurse-led hypertension interventions in East Africa provided evidence that nurse-led interventions are effective in screening, diagnosis, and treatment of hypertension [27].

In one integrative review on nurse-led care interventions for high blood pressure control in Uganda, there was strong evidence to support nurse-led care interventions in the control of high blood pressure [28].

In Uganda, screening and management of ASCVD and hypertension among diabetic patients is a doctor’s role that takes place at health center IV, district, and regional referral hospitals, yet it is the nurses who spend most of the time with the patients and are readily available.

Therefore, the scarce doctors who are also involved in communicable disease care are stretched to provide timely and comprehensive ASCVD risk and hypertension screening and primary prevention in the community. This leads to patients presenting late with established cardiovascular disease for secondary care that is very expensive and fatal.

Improvement in diabetes, hypertension, and ASCVD care requires the transformation of the health system rather than a reactionary approach. Therefore, Wagner’s Chronic Care Model that improves care in health systems at community, organization practice, and patient levels will be adapted in this trial [29].

To our knowledge, no trial using the chronic care model has been conducted to study the effects of a nurse-led lifestyle choice and coaching intervention on systolic blood pressure among patients with type 2 diabetes with high ASCVD risk in Uganda.

### Objectives {7}

The aim of this study is to evaluate the effects of a nurse-led lifestyle choice and coaching intervention on systolic blood pressure and ASCVD risk factor profile among patients with type 2 diabetes. We hypothesize that a nurse-led lifestyle choice and coaching intervention reduces systolic blood pressure and ASCVD risk among type 2 diabetic patients with a high ASCVD risk.

### Trial design {8}

**Fig. 1:**
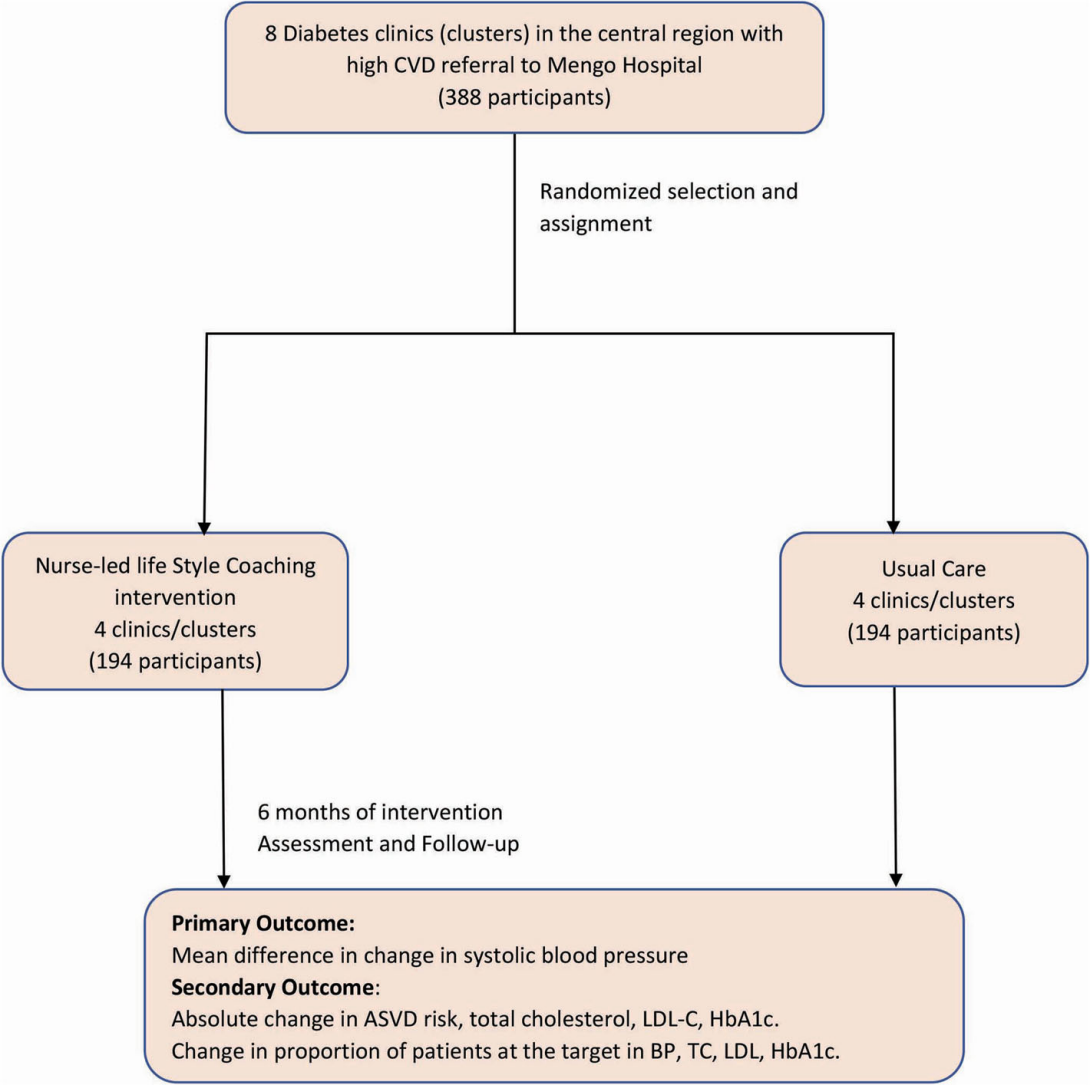
Study design. CVD, cardiovascular disease; ASCVD, atherosclerotic cardiovascular disease; LDL-C, low-density lipoprotein cholesterol

This is a cluster-randomized study with a parallel design in eight [8] diabetes clinics in the central region that usually refer patients with cardiovascular complications to Mengo Hospital as illustrated in Fig. 1. Consented patients with a high ASCVD risk score greater than 7.5% will be eligible for the study. The health facilities include Entebbe Grade B, Wakiso Health Centre IV, Kasangati Health Center IV, Mityana Hospital, Kawolo Hospitals, Mengo Hospital, Naguru Hospital, and Mpigi Health Center IV. These will form fixed clusters for randomization into intervention and control arms. Patients or the public were not involved in the study design.

## Methods: participants, interventions, and outcomes

### Study setting {9}

Out of the 10 health facilities in Central Uganda that refer type 2 diabetic patients with cardiovascular disease to Mengo Hospital, eight [8] clinics which referred most patients in the preceding year were selected for the study. The facilities have nurses, clinical officers, and doctors who provide hypertension and ASCVD care with no formal treatment protocols. They were selected to allow for comparison between a nurse-led intervention and a usual care, which is only possible in health center IV and district hospitals. Eligible diabetic patients will be recruited from Entebbe Grade B Hospital, Mengo Hospital, Naguru Hospital, and Kasangati Health Center IV, which are urban health facilities, and Wakiso Health Center IV, Mpigi Health Center IV, Mityana Hospital, and Kawolo Hospital, which are peri-urban health facilities. These facilities run a weekly diabetes clinic where 50–120 patients are treated according to the size of the health facility. Approximately 250–600 patients with type 2 diabetes attend these clinics per week. The facilities have well-organized manual patients’ registers. Thus, intervention in these facilities will be handy in the prevention and management of hypertension and ASCVD among patients with type 2 diabetes.

### Eligibility criteria {10}

The inclusion criteria for study health facilities are as follows:

1. The study health facility must be running a regular outpatient diabetes clinic.
2. Must be located in an urban or peri-urban area in the central region.
3. The diabetes clinic must be having at least 85 outpatient visits per week to ensure the recruitment of enough participants.
4. The minimum distance between health facilities should be more than 6 km to minimize contamination.
5. The health facilities should not share health professionals to minimize intervention bias.
6. The diabetes clinic must be run by nurses and clinical officers or medical officers with well-maintained records and dispensary.

The eligibility criteria for study participants are as follows:

- Inclusion criteria:

1. Adult men and women with type 2 diabetes aged 40–79 years with a high ASCVD risk score of at least 7.5% as calculated with the pooled cohort risk equations
2. Willing to provide informed consent
3. Asymptomatic for ASCVD (those without a history of non-fatal myocardial infarction, stroke, heart failure, percutaneous coronary intervention, coronary artery bypass surgery, or current atrial fibrillation)

- Exclusion criteria:
  1. Pregnant women (pooled cohort equation has not been validated in pregnancy)
  2. Those with other comorbidities such as chronic kidney disease and liver disease
  3. Patients who do not usually keep appointments, that is, those who have attended clinics only once per year

### Who will take informed consent? {26a}

After confirming eligibility, trained study nurses will obtain written informed consent from potential participants.

### Additional consent provisions for collection and use of participant data and biological specimens {26b}

Blood will be collected, but it will not be used for genetic and molecular analysis in this trial and future.

## Interventions

### Explanation for the choice of comparators {6b}

Out of the 10 health facilities in Central Uganda that refer type 2 diabetic patients with cardiovascular disease to Mengo Hospital, eight [8] clinics which referred most patients in the preceding year were selected for the study. The facilities have nurses, clinical officers, and doctors who provide hypertension and ASCVD care with no formal treatment protocols. Health center IV and above were selected to allow for comparison between a nurse-led intervention and a usual care, which is only possible in health center IVs and district hospitals.

### Intervention description{11a}

#### Nurse-led lifestyle change education and management at the health facility

The purpose of this trial is to evaluate the effects of a nurse-led lifestyle change education, management, and coaching intervention on systolic blood pressure among patients with type 2 diabetes with a high ASCVD risk score of at least 7.5% as determined by the pooled cohort risk equations. The intervention will be targeted at both cluster (group) and individual levels. The clusters will be allocated in a 1:1 ratio between intervention and usual care arms.

**Fig. 2:**
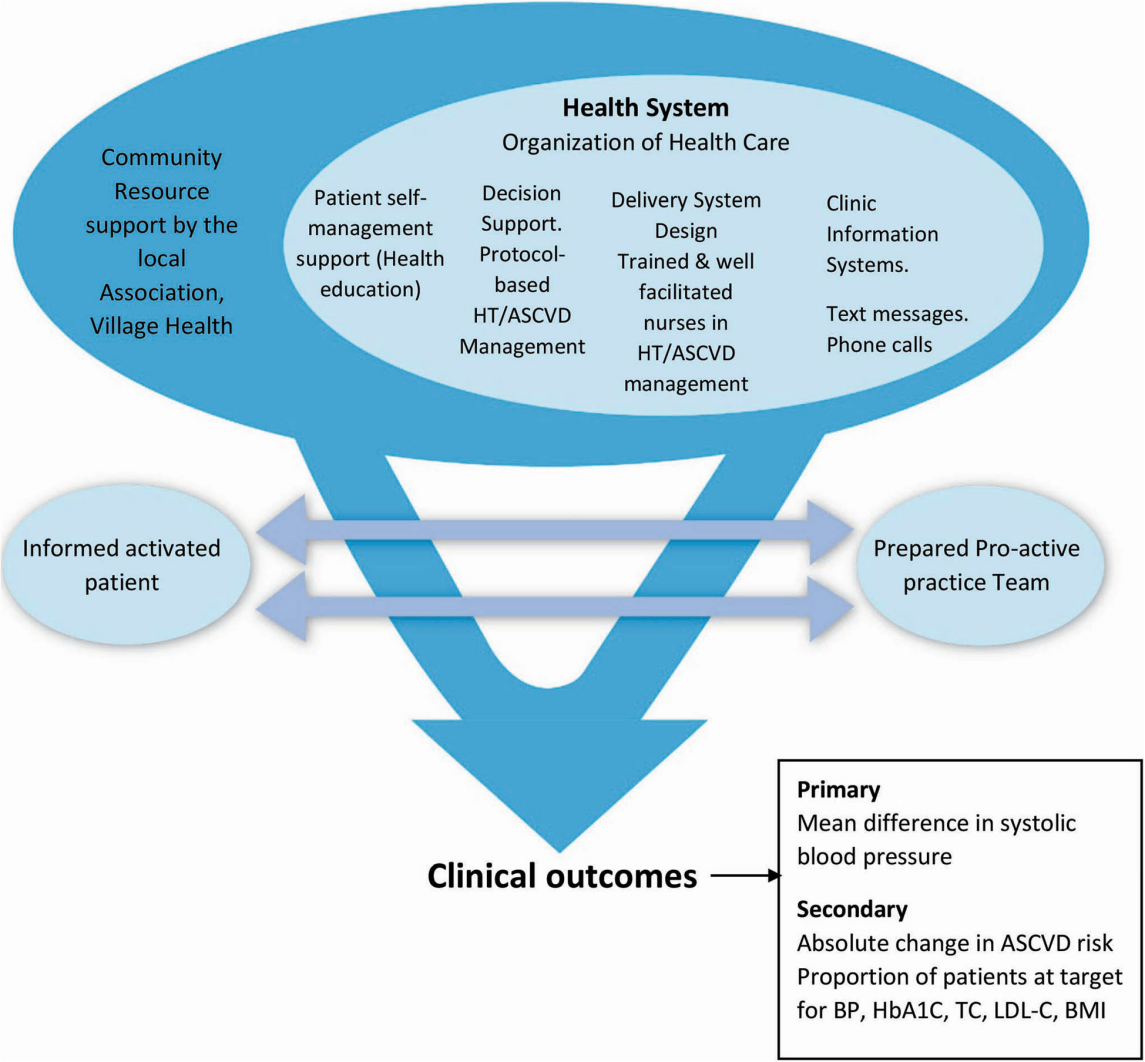
The study conceptual framework. Adapted from Wagner’s Chronic Care Model [29]. HT, hypertension; ASCVD, atherosclerotic cardiovascular disease

Our intervention is based on Wagner’s Chronic Care Model (CCM) as shown in Fig. 2. Wagner’s Chronic Care Model is the best-evidenced strategy to improve diabetes outcomes in the primary care setting [29]. The CCM gives a conceptual framework for reorganizing care from the acute reactive system to a population-based proactively planned care of patients with chronic illnesses such as diabetes, hypertension, and ASCVD [30].

Participants in the intervention group will be subjected to the Nurse-led lifestyle change education, management, and coaching intervention which comprised two components. The first component is the facility-based nurse-led lifestyle change education that is cluster-based, protocol-led hypertension and ASCVD management and treatment adherence counseling that is individualized. The second component is the individualized home-based coaching support.

#### Nurse-led lifestyle change education and management at the health facility

Two nurses from each facility will be identified and trained for 1 day at the study head office in Mengo Hospital. The training will aim to enhance the capacity of the nurses to screen and manage hypertension and ASCVD. They will be trained on ASCVD risk factor assessment with the pooled cohort risk equations, initiation of treatment for hypertension and ASCVD, when and where to refer, and principles of health promotion and behavioral change.

At the end of the training, the nurses will be able to provide group structured health education on ASCVD prevention and management.

The health education sessions will last 30 min, and they will be conducted every 2 months for 6 months. The participants will be educated on the relationship between ASCVD and diabetes, risk factor management, and benefits of medication adherence. The education sessions will be followed by a 15-min question and answer session where participants will be allowed to ask questions. The participants will be provided with an ASCVD leaflet that will be published in both English and the local language. The leaflet will be provided once at the beginning of the trial, but they will be encouraged by the study nurses to come with it every time they come to the health education. The purpose of health education is to produce an informed proactive patient. Improvement in participants’ behavior will be shown with treatment adherence rates and lifestyle change testimonies during sessions. During sessions, participants will elect a leader (peer leader) who will be trained by the study nurses to help in coordinating health education sessions and referral of the critically ill from the community to the health facility.

**Fig. 3:**
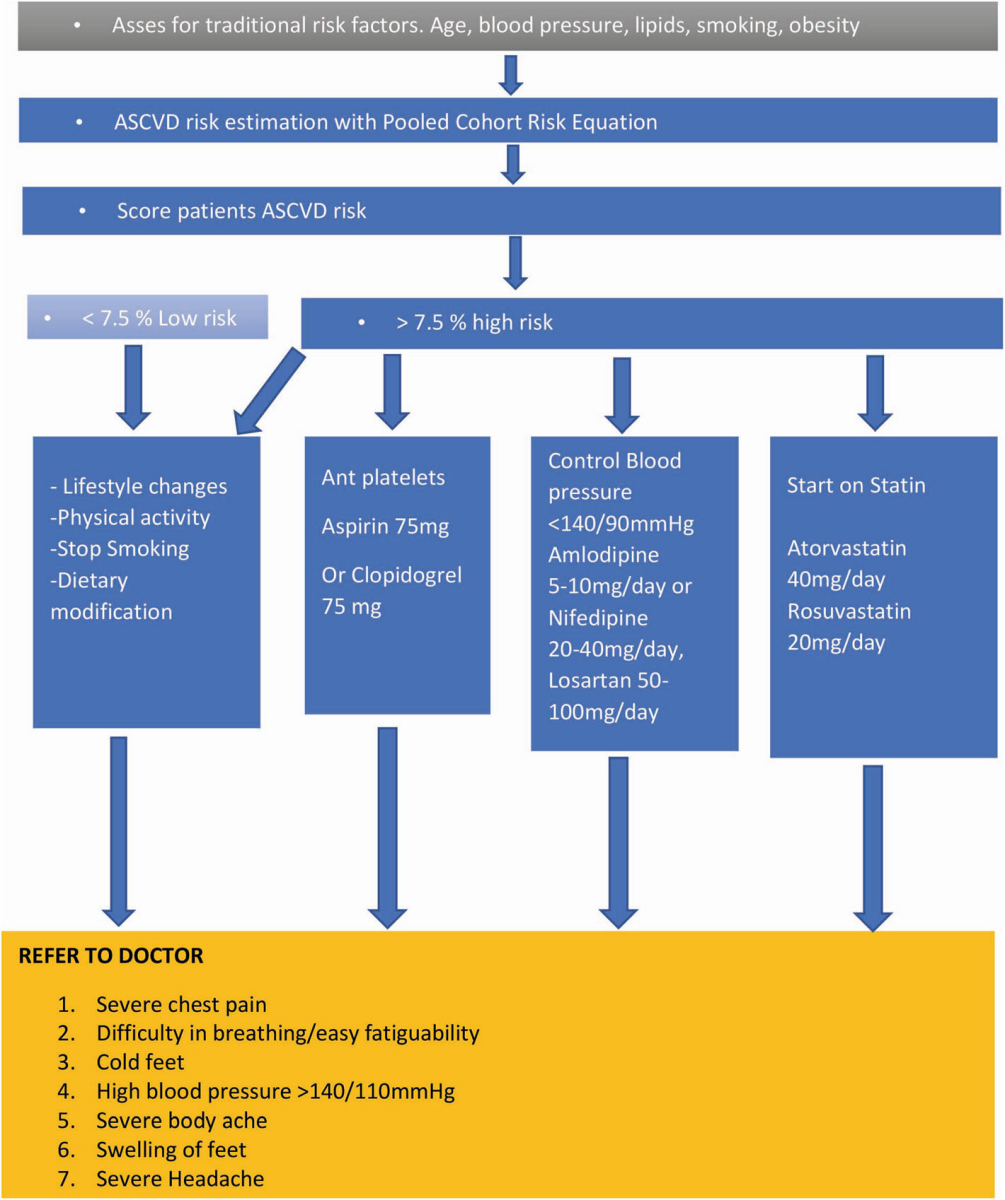
Protocol for ASCVD and hypertension management. ASCVD, atherosclerotic cardiovascular disease

The nurses will provide protocol-based hypertension and ASCVD management as shown in Fig. 3. The protocol has been developed by the principal investigator. The protocol will be used to guide the management and provision of medicine (i.e., lipid-lowering agents, anti-hypertensives). The protocol will give clear guidance on how to determine patient stability and when to refer to a clinical or medical officer in the respective facility or a facility higher. This strategy underpins the task shifting of hypertension and entire ASCVD management from a clinical or medical officer to a well-trained and facilitated nurse with a well health-educated, informed, and supported patient at the center. Therefore, improvement in this capacity will be assessed by the number of patients on appropriate anti-hypertensives, lipid-lowering agents, and referrals.

### Coaching intervention

The second component of the intervention will be the coaching support where individual participants in the intervention group will be given support at home. The study nurses will make individualized telephone calls, send text messages, and operate a 24-h mobile telephone service to answer study participants’ questions and concerns. The phone calls will be structured along the health education topics provided at the health facility. It is anticipated that telephone calls will last for 7 min per participant. Thus, for 194 participants, 22 h of calls will be consumed each time the study participants are called. Participants will be called 3 times during the entire trial period. The participant and the areas discussed will be registered in the call log. Text messages will be sent every after 2 months. The messages will contain topics discussed during the health education talks and in the leaflet. Every time messages are sent, they will be recorded in the text messages’ log.

**Fig. 4:**
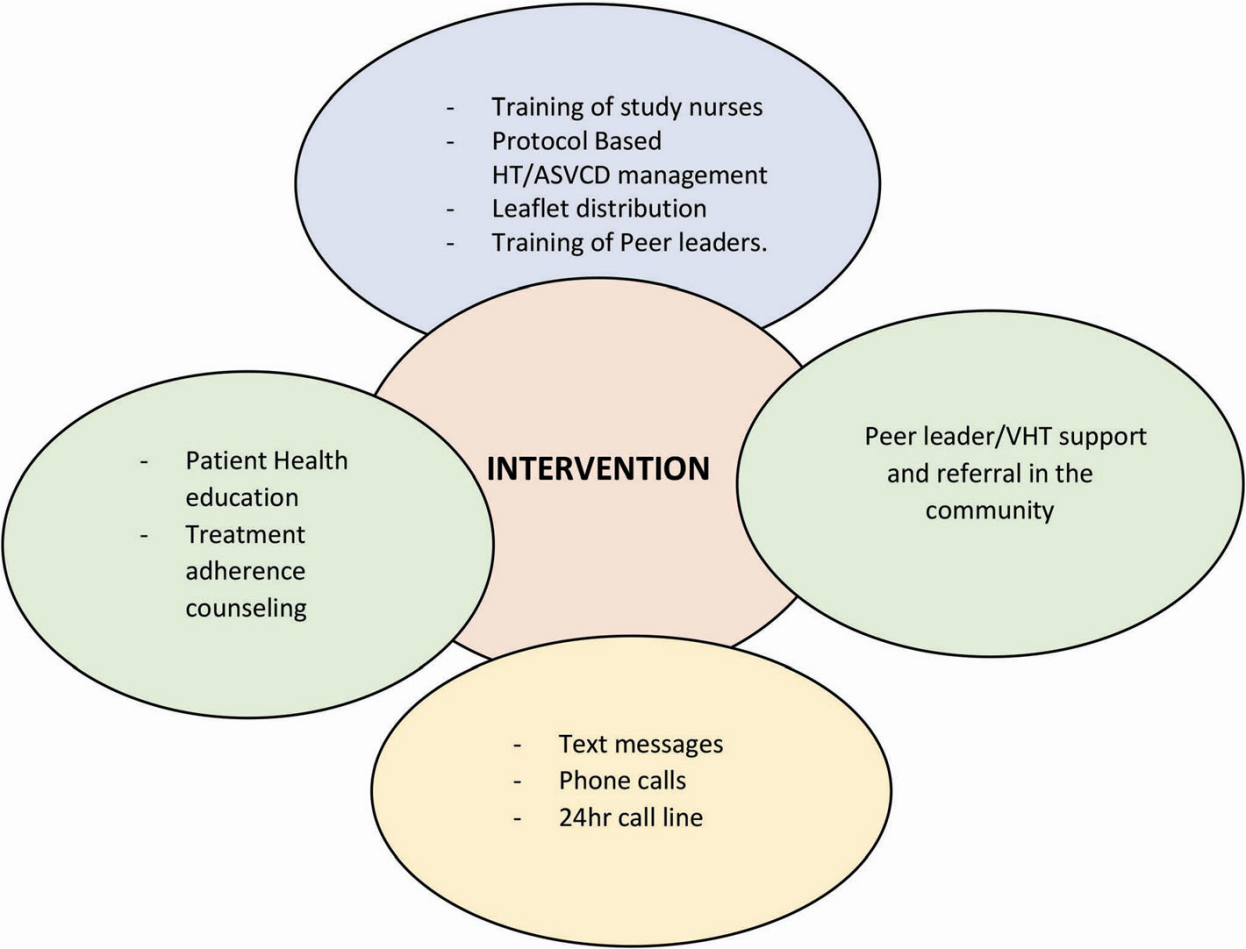
Intervention components. HT, hypertension; ASCVD, atherosclerotic cardiovascular disease; VHT, Village Health Team. 176 × 135 mm (300 × 300 DPI)

The telephone calls and text messages will reinforce individualized hypertension and ASCVD management plan at home. The nurse-patient relationship which is key in the chronic disease care model will be strengthened through calls and text messages. The intervention components are as shown in Fig. 4.

### The usual hypertension/ASCVD care

The control group of the study will comprise the usual care that is the doctor- or clinical officer-led at health centers IV and higher where either patients are referred by nurses or refer themselves with symptoms of ASCVD. In the usual care, patients with diabetes are not subjected to ASCVD risk quantification. Nurses give general health education on diabetes self-care practice which is not structured along with primary hypertension and ASCVD prevention and management. Health education does not build a nurse-patient relationship with a well-educated motivated patient at the center. All patients are given the same management package irrespective of their risk factor profile. Management of hypertension and ASCVD in the health facilities in Uganda is not protocol-based as every facility manages diabetes and hypertension differently. Apart from the general Information Education Communication materials at the clinics, patients are not given information leaflets and are not followed with phone calls and text messages and neither can they call the health workers any time of the day. Patients are followed only in the clinics when they are given review appointments by the doctors or clinical officers. Nurses in these facilities have varied training and skill in diabetes management.

### Criteria for discontinuing or modifying allocated interventions {11b}

Given the fact that the intervention components are some of the routine procedures at the clinics, we anticipate low risk. However, if a participant opts out, or develops an adverse event, she or he will be discontinued from the study.

### Strategies to improve adherence to interventions {11c}

To reduce dropout rates, participants in the intervention arm will be receiving a transport refund. The two monthly calls that are part of the motivation component will also be used to encourage participants to attend follow-up visits. The follow-up log will be used to register visits and also establish those defaulting and their reasons for not attending.

### Relevant concomitant care permitted or prohibited during the trial {11d}

The participants in both intervention and control arms will continue receiving hypertension, diabetes, and ASCVD treatment as deemed appropriate by the health workers in these facilities. The care in the intervention arm will be provided by the trained study nurses according to the study protocol. The care in the control arm will be the usual management provided by the medical officers, clinical officers, and nursing officers.

### Provisions for post-trial care {30}

After the trial, all participants irrespective of their randomization will continue with care in their facilities. The results from the trial will be shared with all the health facilities. Any finding that imparts on treatment outcomes will be shared. Our trial is a low-risk study, and we anticipate low rates of harm. However, in case of an anticipated adverse event, standard operating procedures will be followed.

### Outcomes {12}

#### Primary outcomes

The primary outcome of the trial will be the mean difference in the change in systolic blood pressure between baseline and 6 months between the intervention and usual care groups at individual and cluster levels.

#### Secondary outcomes

The following are the secondary outcomes:

i) Absolute difference and relative change in the predicted 10-year ASCVD risk between the intervention and usual care groups at 6 months
ii) Absolute changes in total cholesterol, low-density lipoprotein cholesterol (LDL-C), glycated hemoglobin, and body mass index at 6 months
iii) Differences between the groups in the change in the proportion of patients reaching treatment goals for systolic blood pressure, total cholesterol, LDL cholesterol, glycated hemoglobin and body mass index at 6 months

### Participant timeline {13}

Participants will be enrolled and assessed following the Standard Protocol Items: Recommendations for Intervention Trials (SPIRIT) schedule as shown in Table 1

**Table 1:** Enrollment and assessment schedule for the study (SPIRIT schedule)

	Study period		
	Enrollment	Allocation	Post-allocation
Time point		0	T1 = 6 months
Enrolment:			
Eligibility screen	X		
Informed consent	X		
Pooled coherent risk equation	X		
Allocation		X	
Intervention:			
Nurse-led lifestyle choice and coaching interventions			
Assessments:			
Baseline variables	- Predicted 10 years ASCVD risk - BMI - Glycated hemoglobin - Systolic blood pressure - Total cholesterol Proportion of patients at target for: - Glycated hemoglobin - Systolic blood pressure - Diastolic blood pressure - Total cholesterol - LDL cholesterol		
Outcome variables			1) Mean change in systolic blood pressure 2) Absolute and relative change in predicted 10-year ASCVD risk 3) Absolute increase in proportion of patients at target for: -Systolic blood pressure -Diastolic blood pressure -Total cholesterol -Glycated hemoglobin -LDL cholesterol

.

### Sample size {14}

To detect an 8.5-mmHg mean difference in systolic pressure between the intervention and control arms, the sample size was calculated basing on a study by Rudd et al. [31]. The study evaluated the effects of a nurse-led use of algorithm-based prescribing compared to usual care where there was a mean change in systolic blood pressure of 14.2 mmHg (standard deviation (SD) = 17.2) in the intervention arm and 5.7 mmHg (SD = 18.7) in the control arm [31].

To have an 80% power for the trial at a significance level of 0.05, a 2-tailed test assuming an intracluster correlation coefficient (ICC)of 0.035 for continuous clinical outcomes for studies related to cardiovascular disease and management in primary care practices [32] has been used. Given that we shall have 4 fixed clinics (clusters) per arm, the sample size for each arm has been calculated using the formula for a fixed number of equal-sized clusters as described by Hemming et al. [33]. The sample size for each arm will be 174. Assuming a 10% loss to follow-up over the 6 months, the final sample size for each arm will be 193.3 rounded to 194 with each cluster having 49 participants. We recognize that the study may not be powered enough for the secondary outcomes since the sample size has been calculated based on the primary outcome.

### Recruitment {15}

The study nurses will carry out announcements during clinic days to recruit patients into the study.

Patients’ files and the clinics’ registers will be reviewed to identify eligible study participants. A list of these will be generated from which they will be selected consecutively.

The study nurses will provide potential study participants with a copy of the informed consent in all study clinics and approved protocol-specific education material in the intervention clinics.

## Assignment of interventions: allocation

### Sequence generation {16a}

The unit of randomization will be the diabetes clinics (clusters) attended by the participants whose ASCVD score has been determined using the pooled cohort risk equations. To ensure the balancing of the arms, the clinics will be paired according to whether they are urban or peri-urban. The urban clinics will comprise Mengo Hospital, Naguru hospital, Entebbe Regional Referral Hospital, and Kasangati Health Center IV while the peri-urban will include Mityana Hospital, Wakiso Health Center IV, Kawolo Hospital, and Mpigi Health Center IV. Two pairs of urban and peri-urban clinics will be formed. These will be randomly allocated in a 1:1 ratio to intervention or control arms using a computer-generated random sequence developed by a statistician uninvolved in the study.

### Concealment mechanism {16b}

An independent statistician will carry out the randomization, and the sequence numbers will be kept in sealed opaque envelopes away from the study clinics. This will ensure the independence of the group allocation from data collection and analysis procedures. The participants who meet the eligibility criteria will be consented. These will have their baseline assessments (*t*_o_) done. Allocation disclosure will occur after baseline measurements in the presence of the study nurses and the participants.

### Implementation {16c}

The allocation and assignment to the intervention will be at the level of the health facilities, and it will be done by the independent statistician.

After confirming eligibility, trained study nurses will obtain written informed consent and collect baseline data that will be filled in the data collection forms.

## Assignment of interventions: blinding

### Who will be blinded {17a}

Due to the type of intervention, blinding of the participants and study nurses will not be possible at data collection, monitoring, and management. The statistician conducting the data analysis will be blinded till completion.

### Procedure for unblinding if needed {17b}

Facility and group allocation will be revealed by the principal investigator to the statistician after completion of data analysis.

## Data collection and management

### Plans for assessment and collection of outcomes {18a}

After confirming eligibility, trained study nurses will obtain written informed consent and collect data on socio-demographic characteristics and treatment history that will be filled in the data collection forms. Baseline data on systolic blood pressure, total cholesterol, high-density lipoprotein cholesterol, low-density lipoprotein cholesterol, glycated hemoglobin, body mass index, and 10-year predicted ASCVD risk score will be collected and filled in the data collection forms.

Study nurses will measure the blood pressure using an automated OMRON® digital blood pressure machine with an appropriate cuff size placed on the upper arm with the bladder of the cuff centered over the brachial artery. An average of three blood pressure measurements will be obtained.

Weight will be measured to the nearest 0.1 kg with the subject standing motionless on the bathroom weighing scale.

Height will be measured to the nearest 0.1 cm with the subject standing in an erect position against a vertical scale or portable stadiometer and with the head positioned so that the top of the external auditory meatus is in level with the inferior margin of the bony orbit.

Body mass index (BMI) will be calculated as weight in kilogram divided by squared height in meters. Conventional BMI cutoffs will be used to classify the study population into underweight (BMI < 18.5 kg/m^2^), normal BMI (≥ 18.5 < 25 kg/m^2^, overweight BMI > 25 to < 30 kg/m^2^), and obese > 30 kg/m^2^.

Waist and hip circumferences will be measured twice to the nearest centimeter using a non-stretchable measuring tape, and the mean values will be used for subsequent analysis.

Waist circumference (WC) will be measured halfway between the lowest rib and the iliac crest with the subject standing at the end of gentle inspiration. The hip circumference (HC) will be measured at the level of the greater trochanters. The waist to hip ratio (WHR) and the waist to height ratio (WHtr) will be computed for each participant.

After a 12-h fasting, standard calorimetric methods will be used to assay fasting plasma glucose, total cholesterol, triglycerides(TG), and high-density lipoprotein cholesterol (HDL-C). Low-density lipoprotein cholesterol will be calculated using the Friedwald formula when TG levels are equal or less than 4 mmol/l. Low-density lipoprotein cholesterol will be measured directly when TGs are > 4 mmol/l.

Glycated hemoglobin (HbA1C) will be determined by high-performance liquid chromatography (HPLC). All HbA1c values will be converted to the Diabetes Control and Complications Trial (DCCT) standard levels: HbA1c (DCCT) = 0.923 × HbA1c (measured) + 1.345; *R*^2^ = 0.998 [34].

The predicted 10-year ASCVD risk will be calculated by the pooled cohort risk equation [35].

Follow-up visits will be scheduled at 6 months after the baseline visit. Anthropometric measurements, blood pressure, predicted 10-year ASCVD risk, glycated hemoglobin, total cholesterol, and BMI will be measured.

### Plans to promote participant retention and complete follow-up {18b}

To reduce dropout rates, participants will be receiving a transport refund. Periodical calls will be conducted to ensure continual follow-up. A follow-up log will be used to register visits and also establish those not attending and the reasons for defaulting.

### Data management {19}

The study nurses will be educated and trained by the principal investigator on proper data correction and completion of data correction forms in accordance with the trial protocol.

At each health facility, data will be compiled into data collection forms, which will be checked daily by the study nurses for completeness. Data collection forms will be secured in lockers at the health facilities and later sent to the principal investigator quarterly for further management. The principal investigator will send data collection forms quarterly to the statistician. Data collection forms will only bear study identification numbers for confidentiality. Data will be entered into the EPI-INFO program and then exported to SPSS statistical program for analysis by the study statistician.

### Confidentiality {27}

The principal investigator and study nurses will generate codes for the study sites (health facilities). In each facility, the participants will be given a participant identification number in a register. From the health facility codes and participant identification numbers, a study identification number will be generated to be used on the data collection forms and specimen containers to protect their privacy.

The records will be kept in a locked location at the study health facility only accessed by those working on the study.

The participant’s name will not be used in any reports, presentations, or publications resulting from this study. The participant’s names will only be revealed in case of referral for further treatment.

### Plans for collection, laboratory evaluation, and storage of biological specimens for genetic or molecular analysis in this trial/future use {33}

Blood will be collected, but it will not be used for genetic or molecular analysis in this trial or future

## Statistical methods

### Statistical methods for primary and secondary outcomes {20a}

Participant characteristics will be described using simple descriptive characteristics. We will use the mean and standard deviation to describe normally distributed continuous variables and the median and upper and lower limits of the interquartile range to describe non-normally distributed continuous variables. Normality of continuous variables will be assessed by visual inspection of histograms. Categorical data will be presented in counts and percentages.

Data will be analyzed according to the intention-to-treat (ITT) principle.

The primary outcome of the trial is the mean difference in the change in systolic blood pressure between baseline and 6 months and between the intervention and usual care groups at individual and cluster levels. It will be expressed as the mean systolic blood pressure difference between the groups (with 95% CI).

This value will be measured as the difference between matched diabetes clinics (intervention clinics minus control clinics) in systolic blood pressure (systolic blood pressure in the intervention period minus systolic blood pressure in the baseline period). The hypothesis for this study is that a nurse-led lifestyle choice and coaching intervention reduces systolic blood pressure compared to usual care among type 2 diabetic patients with high ASCVD risk.

In this study, the same individuals will be assessed at baseline and follow-up (cohort design), and analysis of covariance (ANCOVA) will be used to analyze each individual’s outcome at follow-up adjusted for that individual’s outcome at baseline with mixed-effects linear regression or generalized estimating equations to assess changes over time in systolic blood pressure as a continuous variable between clusters [36, 37]. A random intercept representing each cluster will be added to each model to account for the intra-cluster correlation (ICC). We shall adjust the analysis models by the balancing factor urban or peri-urban.

Appropriate analyses will be employed to assess the secondary outcomes between study arms. Since sample size calculation for the trial was based on the primary outcome, the lack of significant differences between the arms may be due to the true lack of differences or due to inadequate power. Therefore, secondary outcome interpretation will be cautiously done.

All analyses will be conducted using the SPSS software version 25.

### Interim analyses {21b}

No interim analyses are planned due to the low-risk nature of the study.

### Methods for additional analyses (e.g., subgroup analyses) {20b}

No subgroup analyses will be performed.

### Methods in analysis to handle protocol non-adherence and any statistical methods to handle missing data {20c}

We anticipate missing data at random (MAR), where missingness will not depend on the unobserved data but conditional to the observed data. We shall use observed data to impute missing values for patients with missing data. Multiple imputations will be employed to take uncertainty into account by replacing each missing value with a set of possible values to create multiple imputed values. The number of imputations will be determined by the percentage drop out rate. In this study, we anticipate a 10% drop out; therefore, we are planning 10 imputations. We shall perform sensitivity analysis that will weaken the missing data assumption to assess departures in the primary analysis.

### Plans to give access to the full protocol, participant-level data, and statistical code {31c}

Access to the full protocol, participant-level data, and statistical code will only be possible through authorization by the principal investigator.

## Oversight and monitoring

### Composition of the coordinating center and trial steering committee {5d}

The study coordinating center is at Mengo Hospital. The coordinating center comprised the principal investigator, study coordinator, and data entry assistant. The principal investigator and the study coordinator have created the study trial manual and have established the data collection processes including the standard operating procedures. The principal investigator working in conjunction with the other authors (DK, RW and SB) was charged with the conception of the research question and developed the proposal which was submitted for approval. The principal investigator and study coordinator trained study nurses and provide routine communication to the study health facilities. The study coordinator is in charge of routine communication with the study facilities. The study coordinator prepares the monthly progress report to the Mengo Hospital Ethics Committee. The principal investigator, study coordinator, and data entry assistant are responsible for the data oversight. The study coordinator is in charge of logistical provision to the study facilities. The PI and study coordinator work in conjunction with the study nurses to monitor and report adverse events. An independent trial steering committee to oversee the trial has been formed. The Trial Steering Committee comprised an independent epidemiologist, diabetologist, cardiologist, biostatistician, principal investigator, and study coordinator. The independent trial steering committee oversees the trial. Study nurses identify and recruit potential participants and guide them through the consenting process. The Trial Steering Committee (TSC) meets every after 2 months during the trial. The trial does not have stake holder and public involvement group (SPIG).

### Composition of the data monitoring committee, its role, and reporting structure {21a}

The data will be monitored by the team from the coordinating center.

### Adverse event reporting and harms {22}

The study is largely a low-risk one. However, in case of any adverse event or harm, the study participants will be required to inform the study nurse immediately. If the patient develops an AE, he or she will be discontinued from the study. The study nurse will record the event and will follow the study standard operating procedures on the management of adverse events and harms. The serious adverse event will be reported to the coordinating center in Mengo Hospital.

### Frequency and plans for auditing trial conduct {23}

We have no plans for independent trial auditing.

### Plans for communicating important protocol amendments to relevant parties (e.g., trial participants, ethical committees) {25}

Any protocol amendments that may affect the study design or conduct and patient safety will be submitted to the Mengo Hospital Research Ethics Committee for approval. If approved, this will be communicated to the Uganda National Council of Science and Technology and the study health facilities. The Pan African Trial Registry and the journal publishing the protocol will be appropriately informed.

## Dissemination plans {31a}

We will disseminate the findings from this trial through peer-reviewed publications and local and international scientific meetings. A project report written in non-technical language will be sent to all participants.

## Discussion

Atherosclerotic cardiovascular disease (ASCVD) is a leading cause of morbidity and mortality among patients with type 2 diabetes in Uganda. Hypertension which is a major contributor to ASCVD is highly prevalent, undiagnosed, and poorly controlled. One of the reasons for poor control of hypertension and ASCVD in the primary care setting is the scarcity of doctors [13]. Nurses are readily available and spend more time with the patients and are traditionally well versed in self-care and administration of systematic educational interventions [27]. Thus, there is a need to shift the task of management of hypertension and ASCVD from doctors to nurses. However, there is a paucity of data on nurse-led hypertension management among patients with type 2 diabetes in our setting. To the best of our knowledge, this is the first trial in Uganda that examines the effect of a nurse-led lifestyle choice and coaching intervention on systolic blood pressure among patients with type 2 diabetes with a high ASCVD risk. The intervention is based on Wagner’s chronic care model that has been extensively studied in diabetes care. We believe findings from this trial will be useful in drafting national guidelines on the management of hypertension and ASCVD among patients with type 2 diabetes.

## Trial status

Protocol version 03, 2021 January 19

Recruitment is planned to start on 28 December 2020 and is expected to last for 6 months until 1 July 2021.

## Abbreviations

PACTR: Pan African Trials Registry
ASCVD: Atherosclerotic cardiovascular disease
NHANES: National Health and National Survey
UKPDS: United Kingdom Prospective Diabetes Study
HOT: Hypertension Optimal Treatment
SHEP: Systolic Hypertension in the Elderly Program
Syst-Eur: Systolic Hypertension in Europe
CCM: Chronic care model
SPIRIT: Standard Protocol Items Recommendations for Intervention Trial
ICC: Intracluster correlation coefficient
BMI: Body mass index
WC: Waist circumference
HC: Hip circumference
WHR: Waist to hip ratio
WHtr: Waist to height ratio
HPLC: High-performance liquid chromatography
HbA1C: Glycated hemoglobin
DCCT: Diabetes Control and Complications Trial
ITT: Intention-to-treat
ANCOVA: Analysis of covariance
